# Contribution of IL-1RI Signaling to Protection against *Cryptococcus neoformans* 52D in a Mouse Model of Infection

**DOI:** 10.3389/fimmu.2017.01987

**Published:** 2018-01-19

**Authors:** Mitra Shourian, Ben Ralph, Isabelle Angers, Donald C. Sheppard, Salman T. Qureshi

**Affiliations:** ^1^Division of Experimental Medicine, McGill University, Montreal, QC, Canada; ^2^Meakins-Christie Laboratories, McGill University, Montreal, QC, Canada; ^3^Program in Infectious Diseases and Immunology in Global Health, Centre for Translational Biology, The Research Institute of the McGill University Health Center (RI-MUHC), Montreal, QC, Canada; ^4^Department of Microbiology and Immunology, McGill University, Montreal, QC, Canada; ^5^Program in Translational Research in Respiratory Diseases, Department of Critical Care, The Research Institute of the McGill University Health Center (RI-MUHC), Montreal, QC, Canada; ^6^Department of Medicine, McGill University, Montreal, QC, Canada

**Keywords:** *Cryptococcus neoformans*, fungal pneumonia, interleukin-1, interleukin-1 receptor, lung inflammation, cytokines, macrophage polarization, lymphocyte differentiation

## Abstract

Interleukin-1 alpha (IL-1α) and interleukin-1 beta (IL-1β) are pro-inflammatory cytokines that are induced after *Cryptococcus neoformans* infection and activate the interleukin-1 receptor type I (IL-1RI). To establish the role of IL-1RI signaling in protection against cryptococcal infection, we analyzed wild-type (WT) and IL-1RI-deficient (IL-1RI^−/−^) mice on the BALB/c background. IL-1RI^−/−^ mice had significantly reduced survival compared to WT mice after intratracheal challenge with *C. neoformans* 52D. Microbiological analysis showed a significant increase in the lung and brain fungal burden of IL-1RI^−/−^ compared to WT mice beginning at weeks 1 and 4 postinfection, respectively. Histopathology showed that IL-1RI^−/−^ mice exhibit greater airway epithelial mucus secretion and prominent eosinophilic crystals that were absent in WT mice. Susceptibility of IL-1RI^−/−^ mice was associated with significant induction of a Th2-biased immune response characterized by pulmonary eosinophilia, M2 macrophage polarization, and recruitment of CD4^+^ IL-13^+^ T cells. Expression of pro-inflammatory [IL-1α, IL-1β, TNFα, and monocyte chemoattractant protein 1 (MCP-1)], Th1-associated (IFNγ), and Th17-associated (IL-17A) cytokines was significantly reduced in IL-1RI^−/−^ lungs compared to WT. WT mice also had higher expression of KC/CXCL1 and sustained neutrophil recruitment to the lung; however, antibody-mediated depletion of these cells showed that they were dispensable for lung fungal clearance. In conclusion, our data indicate that IL-1RI signaling is required to activate a complex series of innate and adaptive immune responses that collectively enhance host defense and survival after *C. neoformans* 52D infection in BALB/c mice.

## Introduction

*Cryptococcus neoformans* is an encapsulated yeast that is estimated to cause approximately 223,000 cases of meningitis each year and is responsible for 15% of AIDS-related deaths (1). In healthy individuals, inhalation of infectious propagules is usually contained in the lung, but among those with a defective immune response, uncontrolled replication may result in dissemination to other parts of the body with a tropism for the brain (2, 3). Severe cryptococcal disease occurs primarily in patients with uncontrolled HIV/AIDS and is also found in solid organ transplant recipients, those receiving exogenous immunosuppression, patients with primary or acquired immunodeficiency, and increasingly among immunologically normal hosts (4–7).

The pattern of cytokine expression is a crucial determinant of the pathogenesis of cryptococcal infection (3, 8–11). Th1-type cytokines [interleukin (IL)-12 and IFNγ] promote phagocytosis by dendritic cells (DCs) and polarize macrophages toward a classically activated phenotype (M1), thereby increasing fungal clearance (12–15). On the other hand, Th2-type cytokines (IL-4, IL-5, and IL-13) are associated with a significant eosinophil chemotaxis to the lungs and induction of alternatively activated (M2) macrophages that facilitate cryptococcal proliferation and dissemination (16–18). There is some evidence that Th17-type cytokines (IL-17A and IL-23) contribute to protection against infection with wild-type (WT) *C. neoformans*; however, they appear to be less effective compared to Th1-type cytokines (19–23). Inhibition of IL-17A expression or signaling had no significant effect on M1 macrophage polarization, resolution of infection, or survival in mice infected with *C. neoformans* H99 that has been engineered to express IFNγ (24, 25). Finally, a prospective analysis of HIV-infected humans suggested a potential role for IL-17 in the immunopathogenesis of cryptococcal meningitis; however, further studies are required to confirm this hypothesis (26).

The mechanisms that initiate and regulate the innate immune response against *C. neoformans* infection are not fully understood. The interaction of *C. neoformans* with host cells triggers production of several pro-inflammatory cytokines including TNFα, IL-6, and IL-1 (27–30). Both interleukin-1 alpha (IL-1α) and interleukin-1 beta (IL-1β) are induced during cryptococcal infection *in vitro* (27, 28, 31–34) and *in vivo* (35–40) in a NLRP3-dependent manner, and internalization of opsonized encapsulated cryptococci has been shown to activate the canonical NLRP3–ASC–caspase-1 and non-canonical NLRP3–ASC–caspase-8 inflammasome (34, 41). The magnitude of IL-1 expression between inbred mice with different genetic backgrounds has also been associated with natural resistance or susceptibility to progressive cryptococcal infection (35). After intratracheal infection with *C. neoformans* 52D, the level of IL-1β expression was 11-fold higher in the lungs of resistant SJL/J inbred mice compared to the susceptible C57BL/6 inbred strain. A subsequent analysis of WT and interleukin-1 receptor (IL-1R)-deficient mice on the C57BL/6 genetic background did not identify significant differences in survival or fungal dissemination after intranasal infection with *C. neoformans* H99; however, at day 12 postinfection, the IL-1R^−/−^ mice had a modest elevation of lung fungal burden (37).

Given the essential role for cytokine-mediated inflammation and the evidence for IL-1α and IL-1β induction in response to *C. neoformans*, we hypothesized that the contribution of IL-1R-dependent signaling to host defense may have been underestimated by infection of WT and IL-1R^−/−^ mice on the susceptible C57BL/6 genetic background with a highly virulent *C. neoformans* strain. To test this hypothesis, we performed intratracheal inoculation of inbred BALB/c mice and IL-1R^−/−^ mice on the same genetic background with *C. neoformans* 52D and analyzed fungal burden and immune responses at serial time points. This approach was chosen to model the process of natural infection in a relatively resistant host with a moderately virulent cryptococcal strain. Our findings demonstrate that IL-1RI^−/−^ mice had a significantly higher fungal burden in the lungs and brains as well as a significantly higher mortality compared to BALB/c mice. In IL-1RI^−/−^ mice, *C. neoformans* 52D infection was associated with heightened lung eosinophilia, elevated airway mucus secretion, and a greater percentage of M2 macrophages and CD4^+^ Th2 cells along with significantly fewer lung neutrophils, DCs, Th1, and Th17 cells. Taken together, this study shows that IL-1R-dependent signaling contributes to protection against *C. neoformans* 52D infection in BALB/c mice by triggering a complex innate and adaptive immune response and raises the possibility that modulation of this signaling axis could be a potential therapeutic strategy.

## Materials and Methods

### Mice

Inbred BALB/c mice were purchased from Charles River and maintained in our facility. IL-1RI^−/−^ mice were purchased from Jackson Labs and backcrossed to BALB/c for 10 generations. Mice were provided with sterile food and water and cared for according to the Canadian Council on Animal Care guidelines. All experiments were performed using 7- to 9-week-old male and female mice. Mice were humanely euthanized with CO_2_ upon completion of experiments, and every effort was made to minimize suffering. All experimental protocols were reviewed and approved by the McGill University Animal Care Committee.

### *Cryptococcus* *neoformans*

*Cryptococcus neoformans* 52D (ATCC 24067) was grown and maintained on Sabouraud dextrose agar (SDA; BD, Becton Dickinson and Company). To prepare an infectious dose, a single colony was suspended in Sabouraud dextrose broth (BD) and grown to early stationary phase (48 h) at room temperature on a rotator. The stationary phase culture was then washed with sterile phosphate-buffered saline (PBS), counted on a hemocytometer, and diluted to 2 × 10^5^ CFU/ml in sterile PBS. The fungal concentration of the experimental dose was confirmed by plating a dilution of the inoculum on SDA and counting the CFU after 72 h of incubation at room temperature.

### Intratracheal Infection with *C. neoformans*

For intratracheal administration of *C. neoformans*, mice were anesthetized with 150 mg/kg of ketamine (Ayerst Veterinary Laboratories) and 10 mg/kg of xylazine (Bayer) intraperitoneally. A small skin incision was made below the jaw along the trachea, and the underlying glands and muscle were separated. Infection was performed by intratracheal injection of 10^4^ *C. neoformans* in 50 µl PBS through a 22-gauge catheter *via* a 1-ml tuberculin syringe. The incision was closed using the 9-mm EZ clip wound closing kit (Stoelting CO), and mice were monitored daily after surgery.

### Tissue Isolation and CFU Assay

After mice were euthanized with CO_2_, their lungs, spleen, and brain were excised and placed in sterile, ice-cold PBS. Tissues were then homogenized using a glass tube and pestle attached to a mechanical tissue homogenizer (Glas-Col) and plated at various dilutions on SDA. Plates were incubated at 37°C for 72 h, and CFU were counted. For survival analyses, mice were inoculated as stated above and monitored twice daily for up to 110 days postinfection.

### Histopathological Analysis

After euthanasia, lungs were perfused with ice-cold PBS *via* the right ventricle of the heart. Using 10% buffered formalin acetate (Fisher Scientific), the lungs were inflated to a pressure of 25 cm H_2_O and fixed overnight. Subsequently, lungs were embedded in paraffin, sectioned at 5 µm, and stained with hematoxylin–eosin (H&E), periodic acid–Schiff (PAS), or mucicarmine reagents at the Histology Facility of the Goodman Cancer Research Centre (McGill University). Representative photographs of lung sections were taken using a BX51 microscope (Olympus), QICAM Fast 1394 digital charge-coupled device camera (QImaging), and Image-Pro Plus software version 7.0.1.658 (Media Cybernetics).

### Flow Cytometry

Lungs were excised using sterile technique and placed in RPMI (Gibco, Invitrogen) supplemented with 10% fetal bovine serum (Wisent). Subsequently lungs were minced using surgical blades and incubated with 1 mg/ml collagenase (Sigma) at 37°C for 1 h. After incubation, lung pieces were passed through a 16-gauge needle and filtered through a 70-µm cell strainer (BD). Red blood cells were removed using ACK lysis buffer, cells were counted with a hemacytometer using trypan blue dye, and 5 × 10^6^ cells in 100 µl FACS buffer/well were dispensed in 96-well plates. Fc receptors were blocked with the addition of unlabeled anti-CD16/32 antibodies [93; eBioscience (eBio)], and single-cell suspensions were stained with the following fluorescence-conjugated anti-mouse monoclonal antibodies purchased from eBio, BD, and BioLegend: CD45 (30-F11), B220 (RA3-6B2), CD3e (145-2C11), CD4 (GK1.5), CD8 (53-6.7), CD49b (DX5), γδ TCR (GL3), CD11b (M1/70), CD11c (N418), MHCII (M5/114.15.2), Ly6G (1A8), CD86 (GL1), CD80 (16-10A1), CD64 (X54-5/7.1), CD24 (M1/69), SiglecF (E50-2440), CD103 (2E7), Ly6C (HK1.4), and CD206 (C068C2). Non-viable cells were excluded using a fixable viability dye reagent (eBio). Lineage negative cells (Lin^−^) were defined as CD45^+^ cells that did not express any other surface markers in this panel. Data were acquired using a LSRFortessa flow cytometer (BD) and analyzed using Flow Jo software. The absolute number of leukocytes was determined by multiplying the percentage of CD45^+^ cells by the total number of counted cells.

### Intracellular Staining

Lungs were processed as described above, and 5 × 10^6^ cells/well were dispensed in 96-well plates. For cytokine analysis, cells were stimulated for 4 h with phorbol 12-myristate 13-acetate (PMA) and calcium ionophore (ionomycin) in the presence of brefeldin A (GolgiPlug) for the final 3 h. Cells were then washed, blocked with anti-CD16/32 antibodies, and stained with the surface antibodies. Cells were then fixed, permeabilized, and stained with IL-13 (eBio13A), IFNγ (XMG1.2), and IL-17A (17B7). Intracellular staining for Nos2 (CXNFT) was done as described for cytokines without PMA and ionomycin stimulation. Data were acquired using a LSRFortessa flow cytometer with gating determined by fluorescence-minus-one controls and analyzed using FlowJo software.

### Total Lung Cytokine and Chemokine Production

Mice were euthanized and lungs flushed with 10 ml of ice-cold PBS. Whole lungs were homogenized in 2 ml PBS with Halt protease and phosphatase inhibitor cocktail (Fisher Scientific) using a sterilized glass tube and pestle attached to a mechanical tissue homogenizer (Glas-Col) and spun at 12,000 rpm for 20 min. Supernatants were collected, and aliquots were stored at −80°C for further analysis. The following cytokines and chemokines in whole-lung protein samples were analyzed using DuoSet enzyme-linked immunosorbent assay kits (R&D Systems): TNFα (DY410), IL-6 (DY406), IL-1β (DY401), IL-1α (DY400), monocyte chemoattractant protein 1 (MCP-1; MJE00), IL-12/IL-23P40 (DY2398), IFNγ (DY485), CXCL1/KC (DY453), IL-17A (DY421), and IL-13 (DY413).

### Neutrophil Depletion

BALB/c mice received an intratracheal inoculum of 1 × 10^4^ CFU of *C. neoformans* strain 52D. Mice were treated with 100 µl of PBS or 200 µg of anti-1A8 antibody (Bio X Cell) in a volume of 100 µl, 1 day before infection and daily during the study. At day 12 postinfection, lungs were excised, and fungal burden was analyzed.

### Statistical Analysis

To test the significance of single comparisons, an unpaired Student’s *t*-test was applied with a threshold *P* ≤ 0.05. For all experiments, the mean and SEM is shown. Survival curves were analyzed by the log-rank test. All statistical analysis was performed with GraphPad Prism software version 6 (GraphPad Software Inc.).

## Results

### IL-1RI^−/−^ Mice Have Impaired Survival and an Increased Fungal Burden in the Lung, Brain, and Spleen following *C. neoformans* 52D Infection

**Figure 1:**
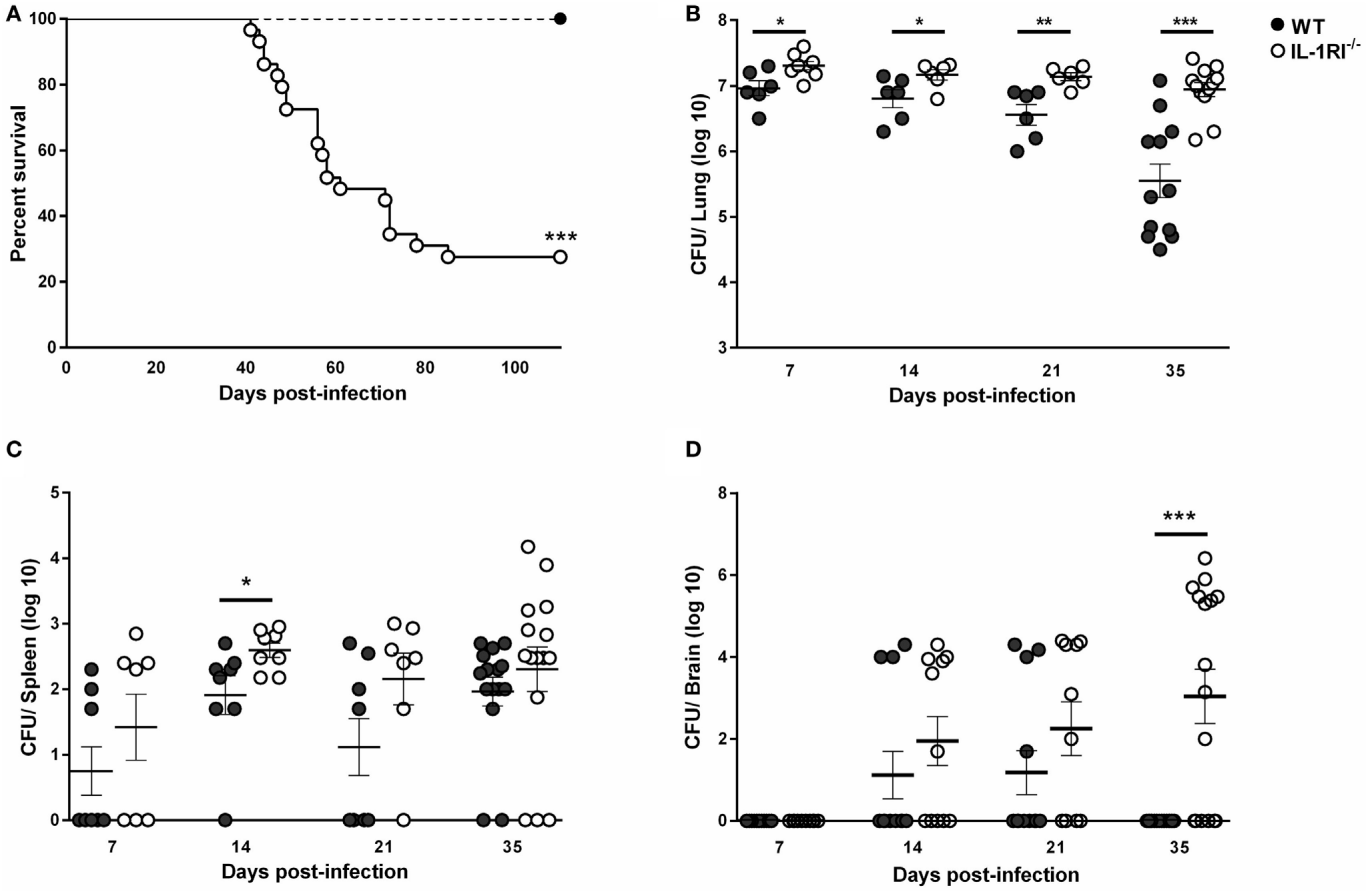
Interleukin-1 receptor type I (IL-1RI) signaling is required for survival and control of fungal burden after infection with *Cryptococcus neoformans* 52D. Wild-type (WT) and IL-1RI-deficient (IL-1RI^−/−^) mice were infected intratracheally with 10^4^ CFU of *C. neoformans* strain 52D. **(A)** Mice were observed for up to 110 days for survival analysis (*n* = 12 mice/strain, using a log-rank test). **(B–D)** Fungal burden in the lung, brain, and spleen at serial time intervals was determined by plating tissue homogenates on Sabouraud dextrose agar. CFU data are shown as mean ± SEM and representative of two independent experiments (*n* = 6–15 mice/strain/time point). *P ≤ 0.05, **P ≤ 0.01, and ***P ≤ 0.001.

To investigate the role of IL-1RI-mediated signaling after *C. neoformans* 52D infection, we constructed IL-1RI^−/−^ mice on the BALB/c background by repeated backcrossing. We challenged mice with *C. neoformans* 52D and measured the survival rate and tissue fungal burden. No deaths were observed in WT mice; however, IL-1RI^−/−^ mice started to die at 40 days postinfection and had a 73% mortality rate at 100 days postinfection (Figure 1A). Microbiological analysis also showed a significant increase of fungal burden in IL-1RI^−/−^ mice compared to the WT strain at all time points tested (Figure 1B). Importantly, a significant difference in lung fungal burden was observed at 7 days postinfection, suggesting that the IL-1RI signaling affects the initial host response to *C. neoformans* infection. At 35 days postinfection, there was almost a 20-fold increase of lung CFU in the IL-1RI^−/−^ compared to the WT strain. Analysis of the spleen showed a trend toward higher CFU in the IL-1RI^−/−^ mice compared to the WT strain that reached statistical significance at day 14 postinfection (Figure 1C). Analysis of the brain showed comparable CFU in both strains at 14 days postinfection; however, at 35 days postinfection, all of the WT mice had cleared the infection, while 10 of 16 (62%) of IL-1RI^−/−^ mice still had detectable fungal growth (Figure 1D). Taken together, these data establish a role for IL-1R-mediated signaling in controlling fungal growth in the lungs and brain, limiting organ dissemination, and increasing survival after *C. neoformans* 52D infection.

### An Altered Pattern of Pulmonary Inflammation Is Present in IL-1RI^−/−^ Lungs following *C. neoformans* 52D Infection

**Figure 2:**
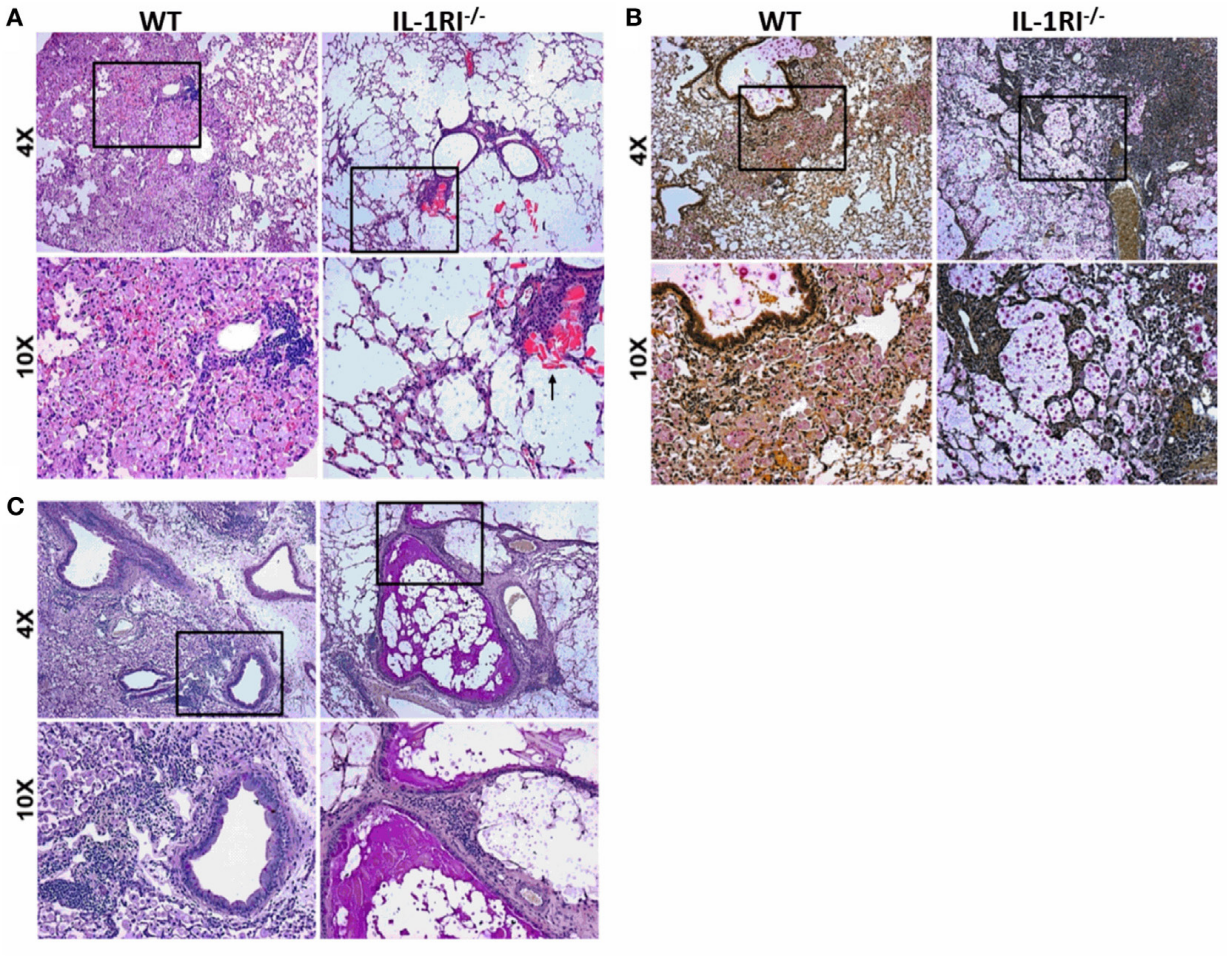
Decreased inflammation in the lungs of interleukin-1 receptor type I-deficient (IL-1RI^−/−^) mice following infection with *Cryptococcus neoformans* 52D. Wild-type (WT) and IL-1RI^−/−^ mice were infected intratracheally with 10^4^ CFU of *C. neoformans* 52D. Lungs were harvested at day 35 postinfection; perfused with phosphate-buffered saline; embedded in paraffin; and stained with hematoxylin–eosin (H&E), mucicarmine, or periodic acid–Schiff (PAS). Representative H&E images **(A)** show a significant reduction of inflammation in IL-1RI^−/−^ compared to WT mice; the black arrow points to eosinophilic crystals in IL-1R^−/−^ lung. Mucicarmine staining **(B)** shows numerous heavily encapsulated extracellular *C. neoformans* in the airspaces of IL-1RI^−/−^ mice compared to WT mice. Representative images of lungs stained with PAS **(C)** show goblet cell hyperplasia and mucus in the airways of infected IL-1RI^−/−^ mice compared to WT mice. Each image is representative of 100 fields examined (*n* = 4 mice/strain from two independent experiments).

The significant differences in survival and fungal burden between WT and IL-1RI^−/−^ mice prompted us to investigate the effect of IL-1RI signaling on lung pathology after infection with *C. neoformans* 52D. Histopathological analysis was conducted at 35 days postinfection to correspond with the greatest difference in fungal burden prior to the onset of mortality (Figures 2A–C). H&E staining revealed that WT mice displayed abundant lung leukocyte infiltration that was almost absent in the IL-1RI^−/−^ strain. Notably, eosinophilic crystals that have been associated with alternatively activated macrophages in *C. neoformans* 52D infection were clearly observed in IL-1RI^−/−^ lung sections but were absent in the WT. Mucicarmine staining of the cryptococcal cell wall showed that most fungi were located within WT phagocytes with only a few visible extracellular organisms in the parenchyma or airways. In contrast, IL-1RI^−/−^ sections showed lung parenchyma that was filled with heavily encapsulated extracellular cryptococci. PAS staining clearly revealed mucus secretion by airway epithelial cells in IL-1RI^−/−^ mice that was not observed in the airways of WT mice. Taken together, this histopathological analysis confirmed the results of the lung fungal burden studies and demonstrated reduced inflammation with signs of Th2 polarization in IL-1RI^−/−^ mice compared to the WT strain.

### Inflammatory Cytokine and Chemokine Production Is Decreased in the Lungs of IL-1RI^−/−^ Mice following *C. neoformans* 52D Infection

**Figure 3:**
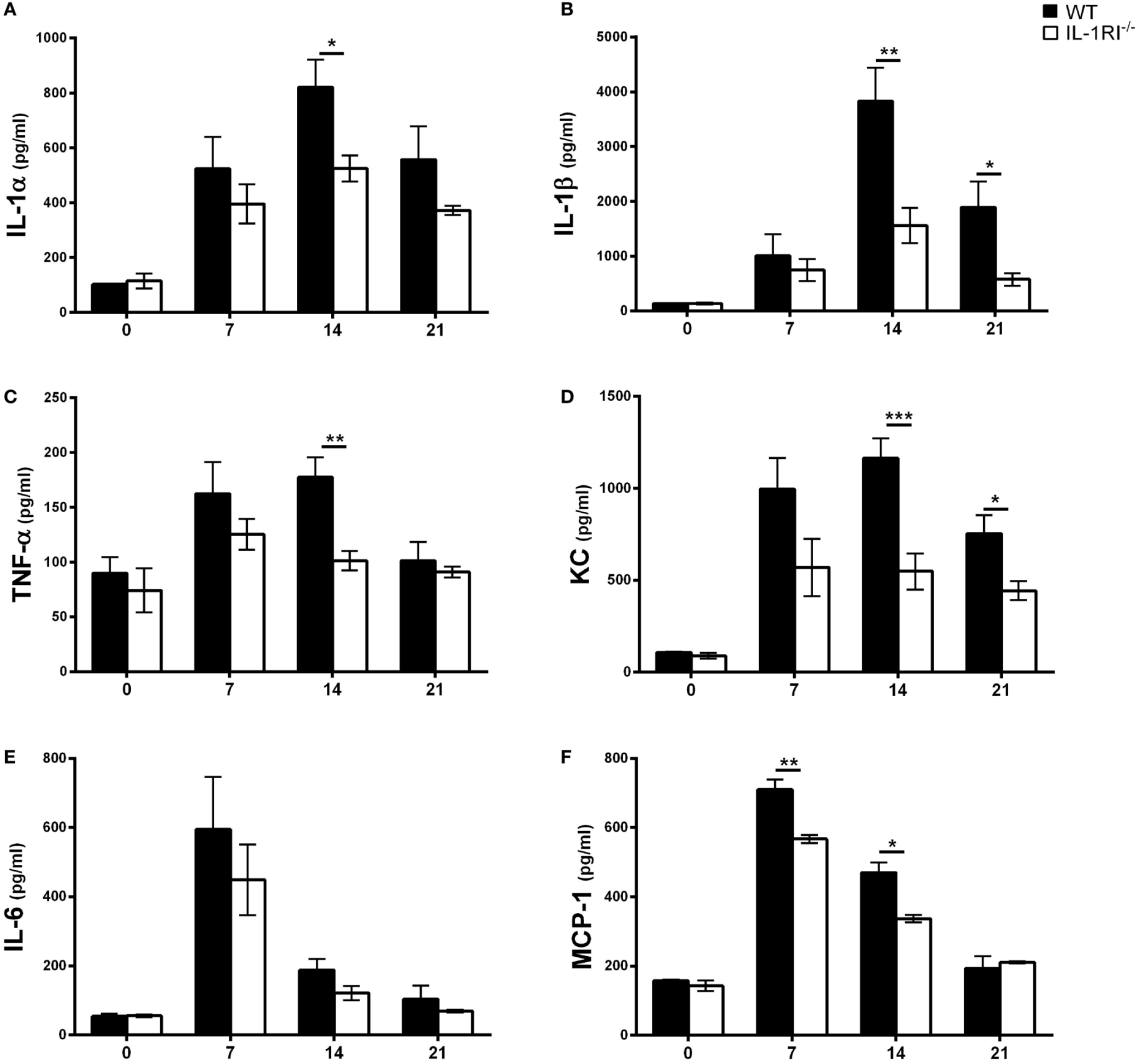

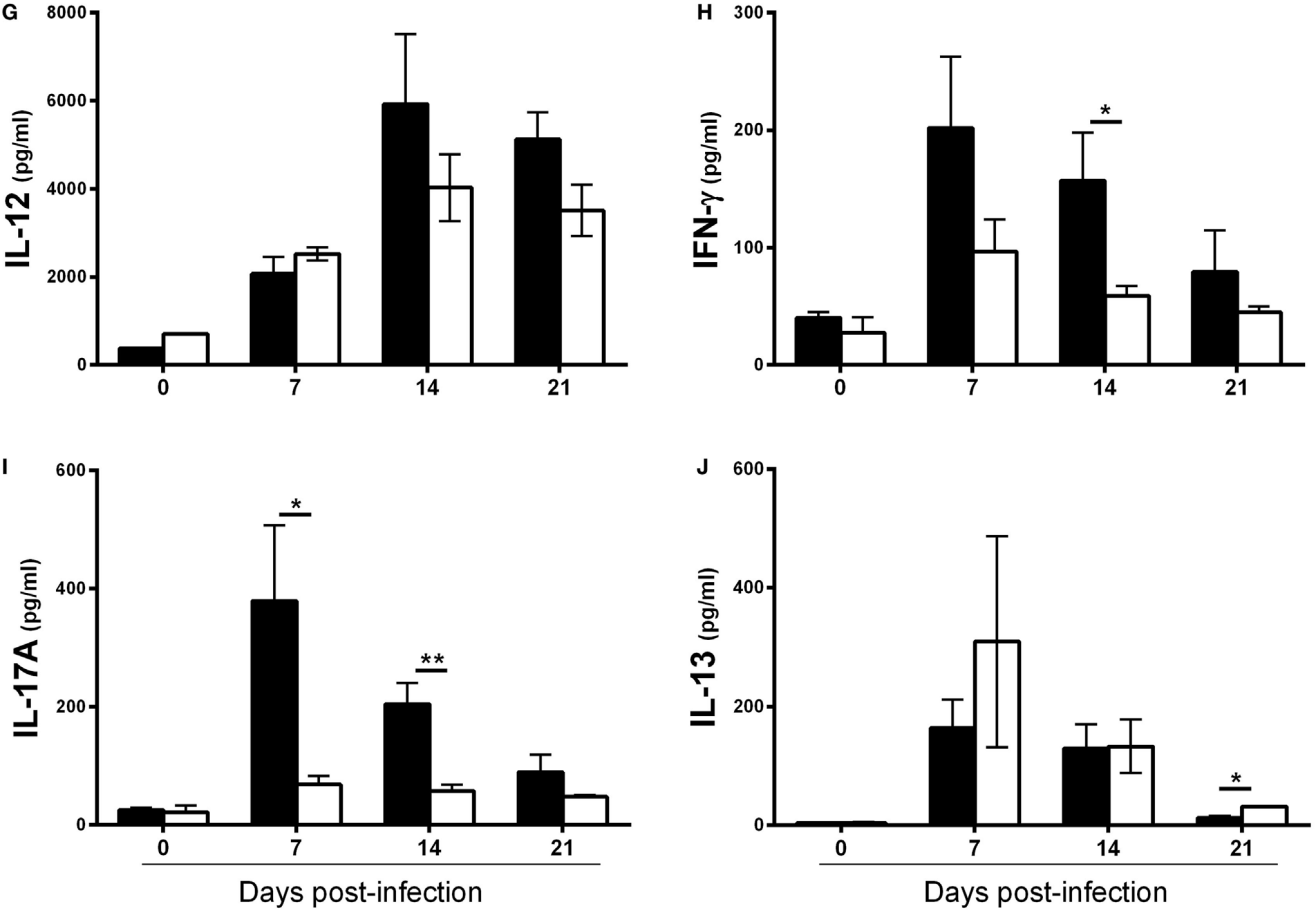
Interleukin-1 receptor type I-deficient (IL-1RI^−/−^) lungs have decreased inflammatory cytokine and chemokine production after *Cryptococcus neoformans* 52D infection. Whole-lung protein was collected at 0, 7, 14, and 21 days postinfection with 10^4^ CFU of *C. neoformans* 52D. Enzyme-linked immunosorbent assay was performed to determine the level of pro-inflammatory cytokines **(A–C, E)**, chemokines **(D, F)**, and Th1/Th2/Th17-type cytokines **(G–J)**. Data are shown as mean ± SEM and representative of two independent experiments (*n* = 4 mice/strain/time point). *P ≤ 0.05, **P ≤ 0.01, and ***P ≤ 0.001. MCP-1, monocyte chemoattractant protein 1.

To determine the effect of IL-1RI signaling on the production of soluble inflammatory mediators, WT and IL-1RI^−/−^ mice were infected with *C. neoformans* 52D, and the concentration of pro-inflammatory cytokines (IL-1α, IL-1β, TNFα, and IL-6), chemokines (MCP-1 and KC), Th1-associated cytokines (IFNγ and IL-12), and representative Th2-associated (IL-13) and Th17-associated (IL-17A) cytokines was measured in whole-lung homogenates at serial time points (Figure 3). No significant differences in the level of these mediators were observed between two strains prior to infection. In WT mice, both IL-1α and IL-1β were produced in the lungs at day 7 postinfection and continued to increase until day 14 postinfection. Compared to WT, IL-1RI^−/−^ mice had significantly lower production of these two cytokines at day 14 postinfection. The production of TNFα, MCP-1, and KC was significantly higher in WT compared to IL-1RI^−/−^ mice at day 14 postinfection. Significantly greater production of IFNγ and IL-17A was also observed in the lungs of WT mice compared to IL-1RI^−/−^ at day 14 postinfection. IL-13 production did not differ between strains at day 7 and day 14 postinfection, although a modest increase was observed in IL-1R^−/−^ mice compared to WT at day 21 postinfection. In summary, BALB/c mice exhibited significantly greater production of pro-inflammatory, Th1, and Th17 cytokines, as well as chemokines, compared to IL-1RI^−/−^ mice; these findings demonstrate a broad effect of IL-1RI signaling on the lung inflammatory response after *C. neoformans* 52D infection.

### IL-1RI^−/−^ Mice Exhibit Reduced Neutrophil and Increased Eosinophil Recruitment to the Lungs following *C. neoformans* 52D Infection

**Figure 4:**
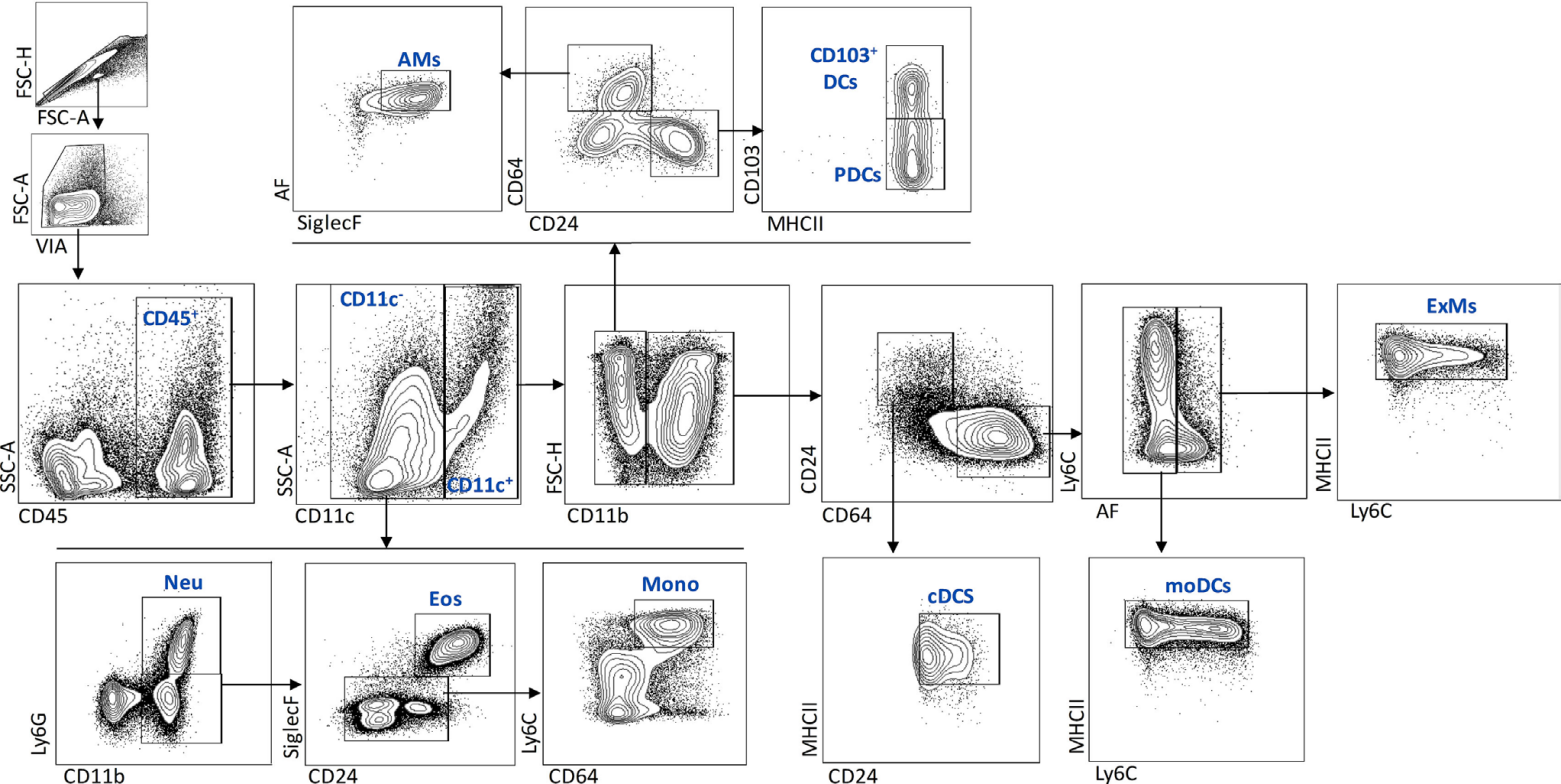
Flow cytometry gating strategy used to identify myeloid cell subsets in the lungs after *Cryptococcus neoformans* 52D infection. Lung cell suspensions were stained with fluorochrome-labeled antibodies and analyzed as described in Section “Materials and Methods.” Representative gating plots for CD45^+^ subsets are shown; neutrophils (CD11c^−^, CD11b^+^, and Ly6G^high^), eosinophils (CD11c^−^, CD11b^+^, Ly6G low/negative, CD24^+^, and SiglecF^+^), monocytes (CD11c^−^, CD11b^+^, Ly6G low/negative, CD64^+^, and Ly6C^high^), AMs (CD11c^+^, CD11b^−^, CD64^+^, SiglecF^+^, and AF^+^), CD103^+^DCs (CD11c^+^, CD11b^−^, CD24^+^, CD103^+^, and MHCII^+^), pDCs (CD11c^+^, CD11b^−^, CD24^+^, CD103^−^, and MHCII^+^), cDCs (CD11c^+^, CD11b^+^, CD64^−^, CD24^+^, and MHCII^+^), mDCs (CD11c^+^, CD11b^+^, CD24^−^, CD64^+^, AF^−^, MHCII^high^, and Ly6C^+/−^), and ExMs (CD11c^+^, CD11b^+^, CD24^−^, CD64^+^, AF^+^, MHCII^+^, and Ly6C^+/−^).

**Figure 5:**
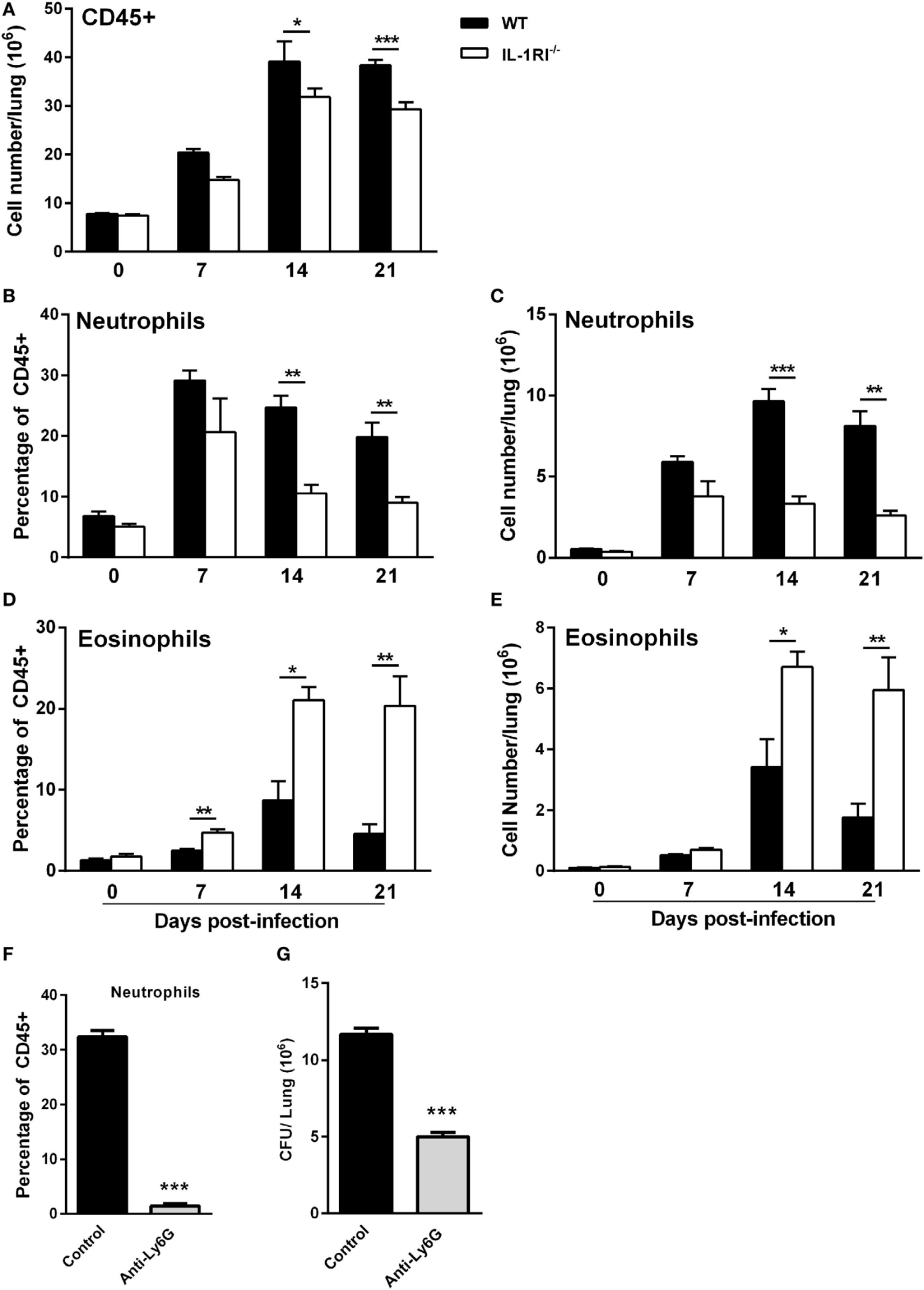
Interleukin-1 receptor type I-deficient (IL-1RI^−/−^) mice have decreased neutrophil and increased eosinophil recruitment to the lungs after *Cryptococcus neoformans* 52D infection. Lung cell suspensions from uninfected and infected wild-type (WT) and IL-1RI^−/−^ mice were stained with fluorochrome-labeled antibodies and analyzed by flow cytometry as described in Section “Materials and Methods.” **(A)** Absolute numbers of total CD45^+^ cells in the lungs at 0, 7, 14, and 21 days postinfection. **(B–E)** Percentage and total number of neutrophils and eosinophils at 0, 7, 14, and 21 days postinfection. Data are shown as mean ± SEM and representative of two independent experiments (*n* = 4 mice/strain/time point). *P ≤ 0.05, **P ≤ 0.01, and ***P ≤ 0.001. **(F,G)** BALB/c mice underwent intratracheal infection with 1 × 10^4^ CFU of *C. neoformans* strain 52D. Mice were treated with phosphate-buffered saline or anti-Ly6G antibody 1 day prior to infection and daily during the study. At 12 days postinfection, lungs were excised for analysis of neutrophil recruitment and CFU. **(F)** The number of neutrophils and **(G)** fungal burden is shown. Data are pooled from two independent experiments and shown as mean ± SEM (*n* = 8 mice/group). ***P ≤ 0.001.

To characterize the effect of IL-1RI signaling on the cellular immune response after *C. neoformans* infection, flow cytometry analysis of whole-lung digests was performed on WT and IL-1RI^−/−^ mice at serial time points postinfection. A comprehensive gating strategy was used for the identification of resident and recruited myeloid cell subsets (Figure 4) (42–44). Prior to infection, no significant difference was observed in the total number of lung leukocytes between the two strains. The total number of CD45^+^ cells peaked at day 14 in both strains; however, it was significantly higher in WT compared to IL-1RI^−/−^ mice at 14 and 21 days postinfection (Figure 5A). At 7 days postinfection neutrophils (CD11c^−^, CD11b^+^, and Ly6G^high^) were the most frequent leukocyte subset in both strains; however, their percentage and total number was significantly higher in the WT compared to the IL-1RI^−/−^ at 14 and 21 days postinfection (Figures 5B,C). Conversely, the percentage and number of lung eosinophils (CD11c^−^, CD11b^+^, Siglec F^+^, and CD24^+^) was significantly higher in IL-1RI^−/−^ mice compared to the WT strain at 14 and 21 days postinfection (Figures 5D,E). These data suggest that IL-1R signaling plays an important role in recruitment of neutrophils during the host response to *C. neoformans* 52D infection. In the absence of IL-1R, mice develop significant and sustained lung eosinophilia that is associated with a higher fungal burden.

To evaluate the functional significance of early and sustained neutrophil recruitment to the lungs of BALB/c mice after infection with *C. neoformans* 52D, the effect of antibody-mediated depletion on tissue fungal burden and lung cell infiltration was characterized. Briefly, WT mice received 200 µg of anti-Ly6G antibody (clone 1A8) in a volume of 100 µl *via* intraperitoneal injection 24 h before infection and daily thereafter. To capture the overall effect of neutrophil depletion during the innate and adaptive phases of immunity, lung fungal burden was determined at 12 days postinfection. Interestingly, this analysis showed that neutrophil-depleted mice had a significantly lower cryptococcal burden in the lungs compared to control mice (Figures 5F–G).

### IL-1RI^−/−^ Mice Recruit Fewer Monocyte-Derived DC and Macrophages to the Lung following *C. neoformans* 52D Infection

**Figure 6:**
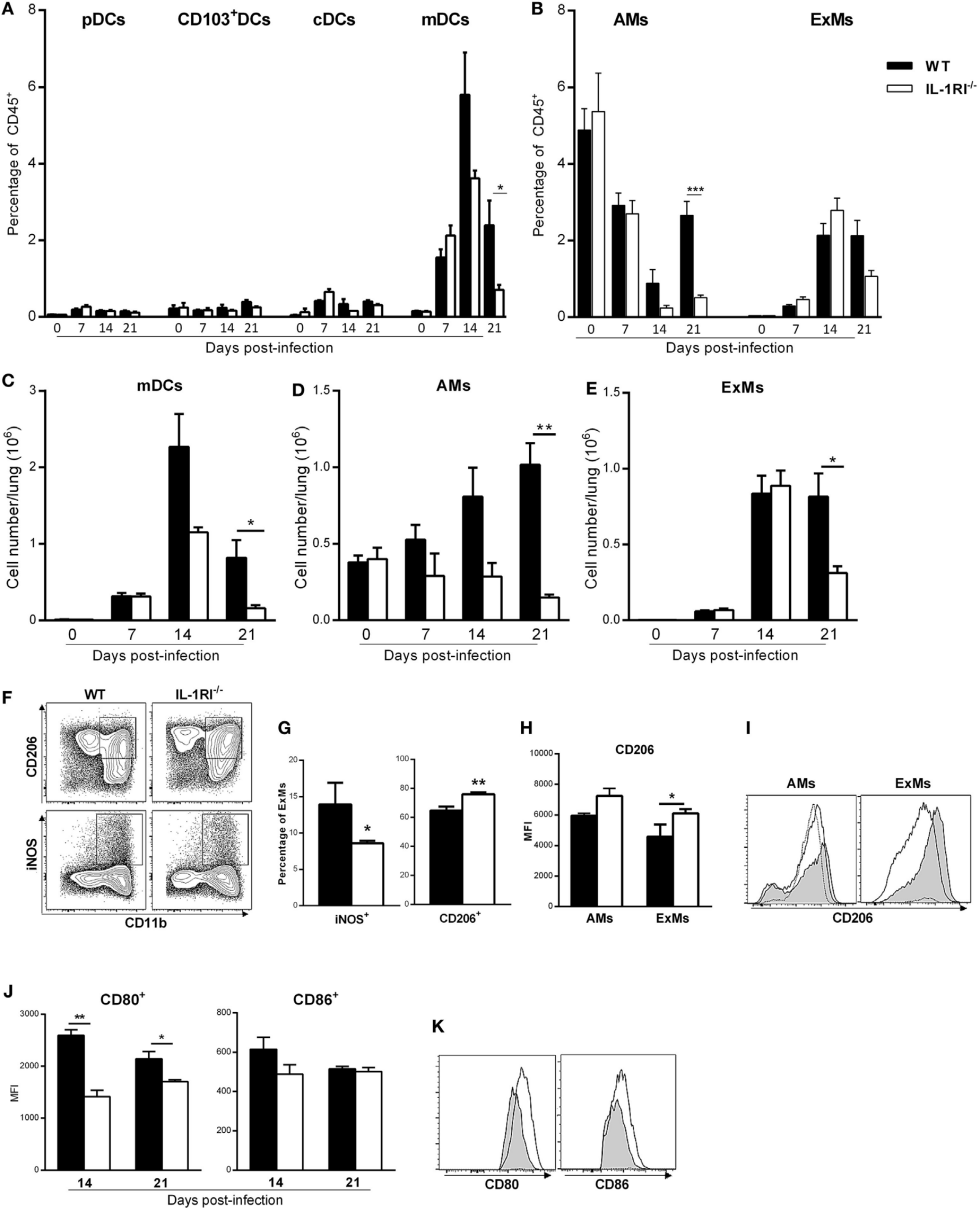
Interleukin-1 receptor type I-deficient (IL-1RI^−/−^) mice have fewer monocyte-derived dendritic cells (DCs) and macrophages in the lungs after *Cryptococcus neoformans* 52D infection. Lung cell suspensions from uninfected and infected mice were stained with fluorochrome-labeled antibodies and analyzed by flow cytometry as described in Section “Materials and Methods.” **(A,B)** Percentage of DC and macrophage subsets at 0, 7, 14, and 21 days postinfection. **(C–E)** Total number of mDCs, AMs, and ExMs at 0, 7, 14, and 21 days postinfection. **(F)** Representative plots of M1 (CD11b^+^, iNOS^+^) and M2 (CD11b^+^, CD206^+^) polarized macrophages in wild-type (WT) and IL-1RI^−/−^ mice at 14 days postinfection. **(G)** Percentage of M1- and M2-polarized macrophages in WT and IL-1RI^−/−^ mice at 14 days postinfection. **(H)** Mean fluorescence intensity (MFI) of CD206 expression on macrophages in WT compared to IL-1RI^−/−^ mice and **(I)** Upregulation of CD206 in AMs and ExMs in IL-1RI^−/−^ compared to WT mice at 14 days postinfection. **(J)** MFI and **(K)** upregulation of CD80- and CD86-positive cells derived from ExMs at 14 days postinfection is shown; **(I,K)** IL-1RI^−/−^, gray filled lines; WT, white filled solid lines; uninfected mice, dashed lines. Data are shown as mean ± SEM and representative of two independent experiments (*n* = 4 mice/strain/time point). *P ≤ 0.05, **P ≤ 0.01, and ***P ≤ 0.001.

Inflammatory monocyte-derived macrophages (ExMs) and DCs are important for protection against *C. neoformans* infection (45, 46). We investigated the effect of IL-1RI signaling on the number of resident and monocyte-derived myeloid cells by harvesting lungs at different times postinfection and analyzing cells by flow cytometry. No significant difference in the percentage of pDCs, CD103^+^ DCs, and CD11b^+^ cDCs was observed in the lungs of BALB/c and IL-1R^−/−^ mice after *C. neoformans* 52D infection; however, at day 21 postinfection, there was a significantly higher percentage and number of AMs in BALB/c compared to IL-1R^−/−^ mice (Figures 6A,B,D). As both monocyte-derived ExMs and DCs are CD11b^+^, CD11c^+^, CD24^−^, MHCII^+^, and CD64^+^, we used autofluorescence to distinguish macrophages from DCs (42, 45, 47) (Figure 4). This analysis showed comparable recruitment of both cell types between the two strains at day 7 postinfection; however, WT mice had a significantly higher number of inflammatory DCs (days 14 and 21) and ExMs (day 21) compared to IL-1RI^−/−^ mice (Figures 6C,E).

The macrophage polarization pattern is also important for protection against cryptococcal infection (8, 48). Classically activated macrophages (M1) that express high levels of pro-inflammatory cytokines and costimulatory molecules, produce high levels of reactive nitrogen and oxygen intermediates, and promote strong IL-12-mediated Th1 responses are efficient killers of *C. neoformans*. In contrast, alternatively activated macrophages (M2) that express chitinase-like 3 (Ym1), found in inflammatory zone (FIZZ1), mannose receptor (CD206), and arginase-1 (Arg1), have reduced pro-inflammatory cytokine secretion and are less microbicidal (3, 9, 42, 43, 47, 49–53). As the number of recruited macrophages peaked at day 14 postinfection in both strains, we characterized polarization at this time point using iNOS and CD206 as representative markers for M1 and M2 macrophages, respectively. At 14 days postinfection, the percentage of M1 macrophages was significantly greater in WT mice compared to IL-1R^−/−^ mice, while the percentage of M2 macrophages was greater in IL-1R^−/−^ compared to WT mice (Figures 6F,G). Notably, IL-1RI^−/−^ macrophages showed greater upregulation of the M2-associated marker CD206 at 14 days postinfection (Figures 6H,I), while WT macrophages displayed higher expression of the M1-associated marker CD80 (43) at 14 and 21 days postinfection (Figures 6J,K). Taken together, these results indicate that IL-1RI signaling has an important role in recruitment of inflammatory DCs and macrophages and increases the ratio of M1/M2-polarized macrophages after *C. neoformans* 52D infection.

### T Cells Are the Predominant Sources of IL-17A and IFNγ in WT Lungs Infected with *C. neoformans* 52D

**Figure 7:**
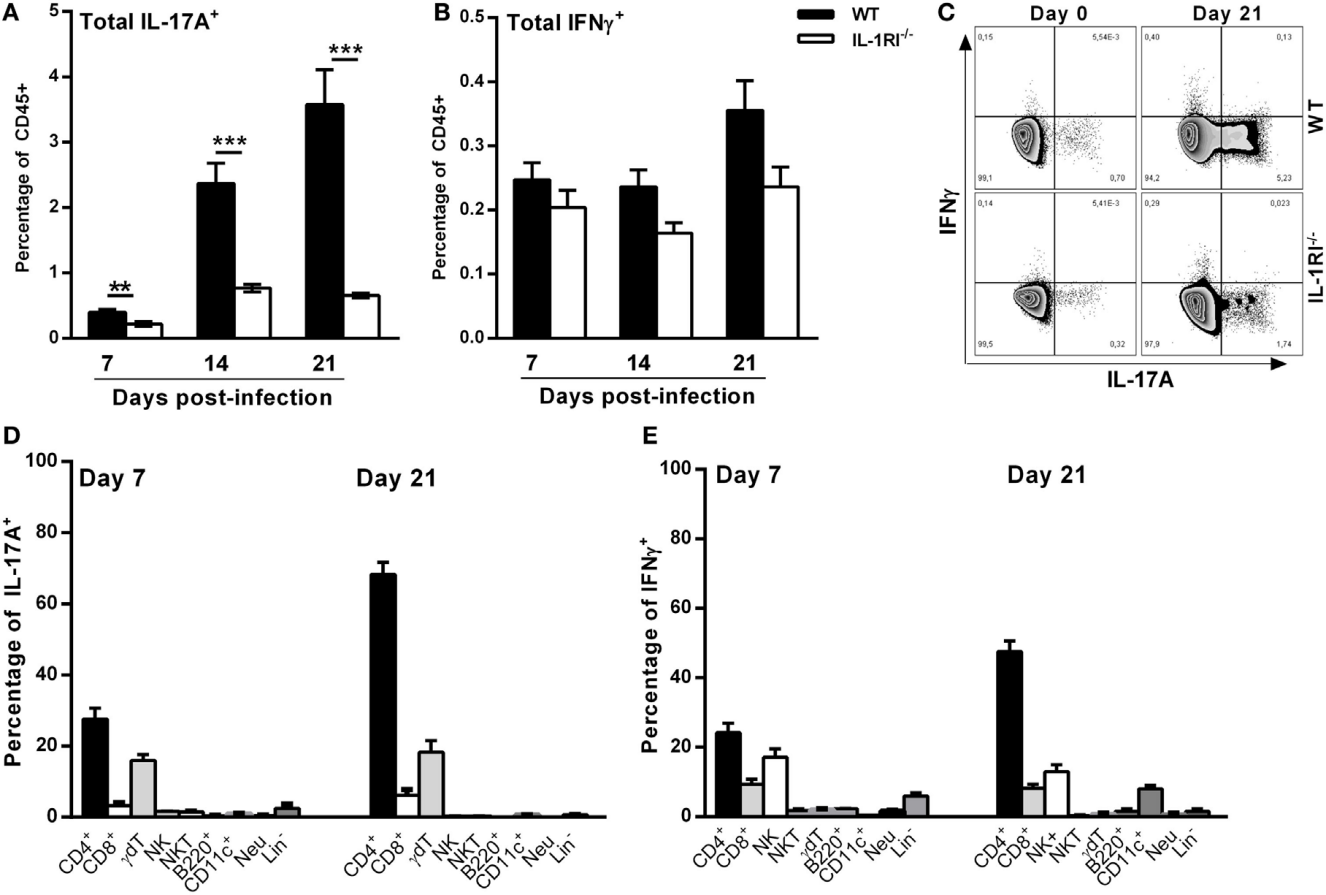
T cells are the predominant sources of interleukin (IL)-17A and IFNγ in BALB/c lungs infected with *Cryptococcus neoformans* 52D. **(A)** Lung cell suspensions from uninfected and infected mice were harvested and restimulated with phorbol 12-myristate 13-acetate (PMA)–ionomycin followed by intracellular staining for IL-17A and IFNγ. **(A,B)** Percentage of total CD45^+^IFNγ^+^ and CD45^+^IL-17A^+^ cells at 7, 14, and 21 days postinfection. **(C)** Representative flow cytometry plots of lung CD45^+^ cells from individual mice harvested at 21 days postinfection. **(D,E)** Percentage of IL-17A- and IFNγ-producing cell types in WT mice at 7 and 21 days postinfection is shown. Data are shown as mean ± SEM and representative of two independent experiments (*n* = 4 mice/strain/time point). **P ≤ 0.01 and ***P ≤ 0.001.

To characterize the mechanism of differential IL-17A and IFNγ expression between WT and IL-1RI^−/−^ lungs, we identified the main sources of these cytokines after *C. neoformans* 52D infection. Compared to IL-1RI^−/−^ mice, WT mice showed significantly more IL-17A-producing cells at 7, 14, and 21 days postinfection and a trend toward a higher number of IFNγ-producing cells at day 21 postinfection (Figures 7A–C). Several immune cell types including CD4^+^ (Th17), CD8^+^ T (Tc17) cells, NK cells, iNKT cells, γδT cells, B cells, ILCs, DCs, and neutrophils have been shown to produce IL-17 during fungal infection (25, 54–56). In our study, at day 7 postinfection, intracellular cytokine staining of WT lymphocytes showed that CD4^+^ and γδT cells were the most common IL-17A^+^ subsets (Figure 7D). A similar pattern was observed at day 21 postinfection with CD4^+^ T cells and γδT cells accounting for 60 and 20%, respectively, of IL-17A^+^ cells. CD4^+^ and CD8^+^ T-cells, NK cells, γδT cells, and neutrophils have been shown to produce IFNγ during fungal infection (57–59). In our study, CD4^+^ T and NK cells were the predominant IFNγ-producing subsets at day 7 and day 21 postinfection (Figure 7E).

### Effect of IL-1RI Signaling on the Lung Lymphocyte Infiltration following *C. neoformans* 52D Infection

**Figure 8:**
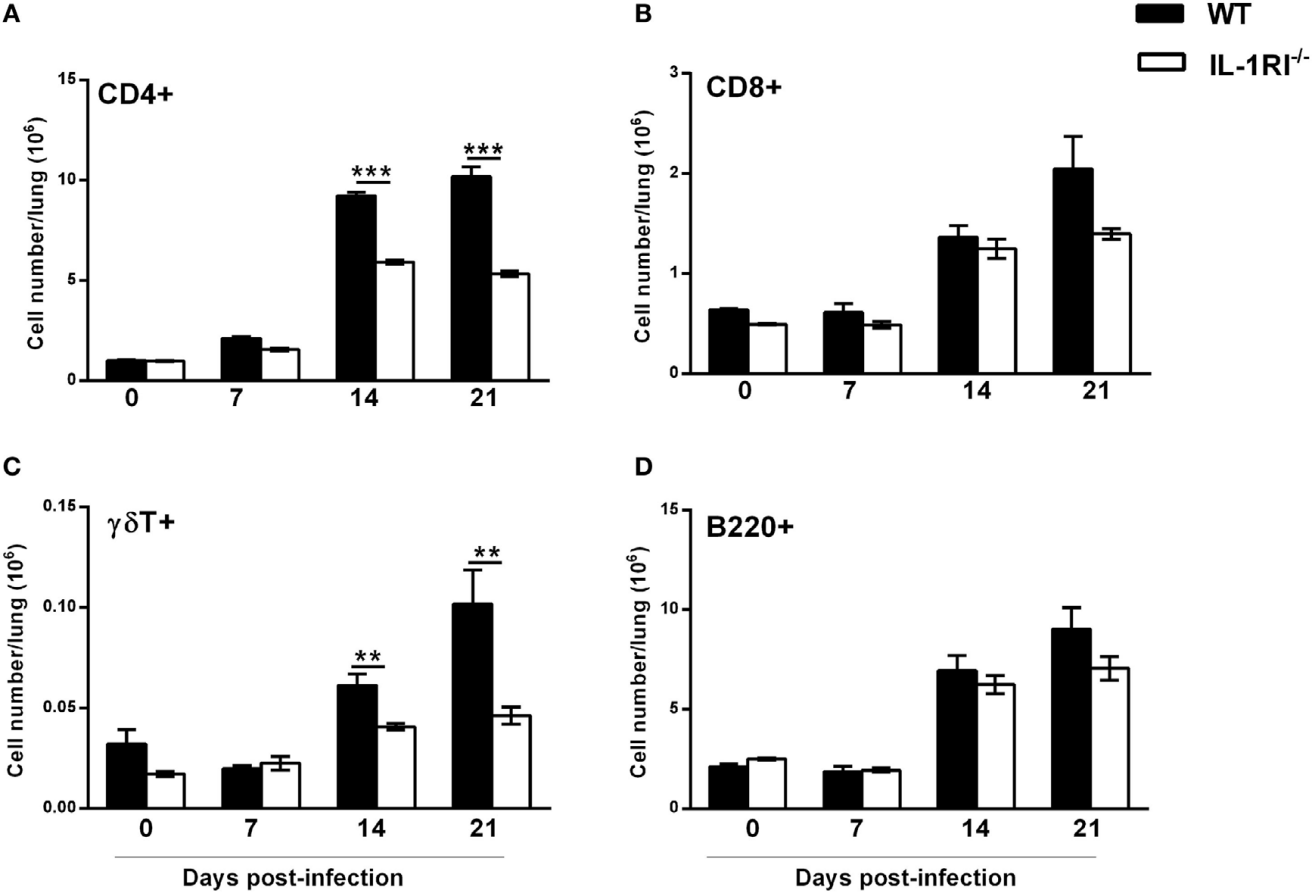
Lungs of interleukin-1 receptor type I-deficient (IL-1RI^−/−^) mice display fewer CD4^+^ and γδT^+^ lymphocytes during the adaptive phase of immunity after *Cryptococcus neoformans* 52D infection. Lung cell suspensions from uninfected and infected mice were stained with fluorochrome-labeled antibodies and analyzed by flow cytometry as described in Section “Materials and Methods.” **(A–D)** Total number of CD3^+^CD4^+^, CD3^+^CD8^+^, CD3^+^γδ^+^, and B220^+^ cells in the lungs at 0, 7, 14, and 21 days postinfection. Data are shown as mean ± SEM and representative of two independent experiments (*n* = 4 mice/strain/time point). **P ≤ 0.01 and ***P ≤ 0.001.

As lymphocytes are necessary for effective clearance of *C. neoformans*, we compared the recruitment of CD4^+^ or CD8^+^ T cells, γδT cells, and B cells to the lungs of WT and IL-1RI^−/−^ mice at different time points after infection. Flow cytometry analysis showed that WT mice recruit a significantly higher number of CD4^+^ cells compared to the IL-1RI^−/−^ strain at 14 and 21 days postinfection (Figure 8A). Recruitment of CD8^+^ T cells was comparable between the two strains at all time points, although WT mice showed a trend toward a higher number of CD8^+^ T cells at day 21 compared to IL-1RI^−/−^ mice (Figure 8B). WT mice demonstrated increased recruitment of γδT cells at day 14 and day 21 postinfection compared to uninfected mice; in contrast, there was no significant increase of this cell type in IL-1RI^−/−^ mice during infection (Figure 8C). No differences in the number of B cells recruited to the lungs during infection were observed between the two strains (Figure 8D). Taken together, this analysis demonstrates that IL-1RI signaling selectively regulates T lymphocyte recruitment to the lungs during the adaptive phase of immunity against *C. neoformans* 52D infection.

### Pulmonary CD4^+^ T Cells from IL-1RI^−/−^ Mice Display Diminished Th17 and Increased Th2 Cytokine Production following *C. neoformans* Infection

**Figure 9:**
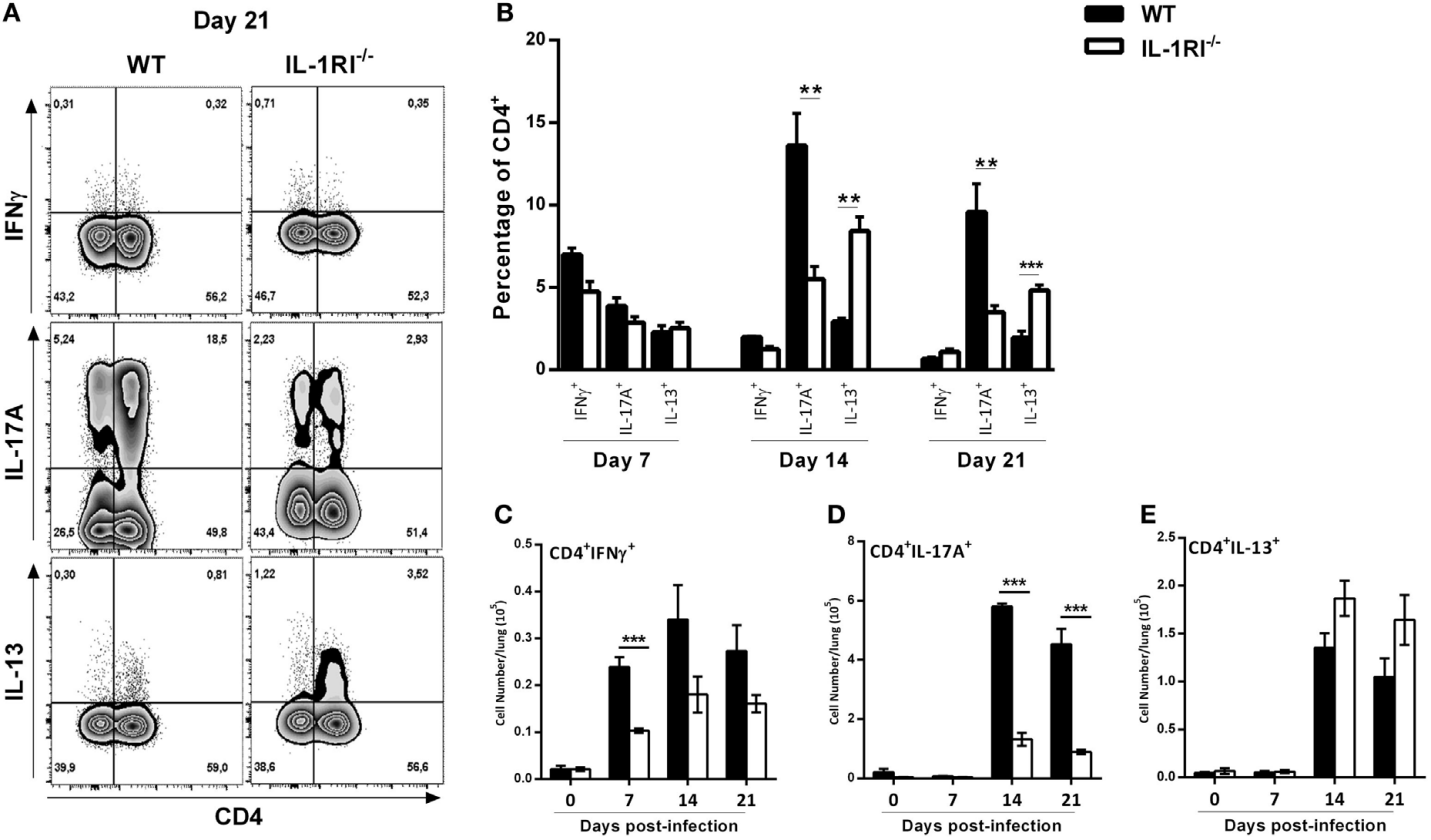
Decreased Th1/Th17 type cytokine expression by CD4^+^ T cells from interleukin-1 receptor type I-deficient (IL-1RI^−/−^) lungs infected with *Cryptococcus neoformans* 52D. **(A)** Representative flow cytometry plots of lung lymphocytes from individual mice harvested at 14 days postinfection and restimulated with phorbol 12-myristate 13-acetate (PMA)–ionomycin, followed by intracellular staining for IFNγ, IL-17A, and IL-13. **(B)** Percentage and **(C–E)** total numbers of CD4^+^IFN^+^, CD4^+^IL-17A^+^ and CD4^+^IL13^+^ cells are shown. Data are shown as mean ± SEM and representative of two independent experiments (*n* = 4 mice/strain/time point). *P ≤ 0.05, **P ≤ 0.01, and ***P ≤ 0.001. WT, wild type.

It has been clearly shown that a Th1/Th17 response is protective and a Th2 response is detrimental, respectively, against *C. neoformans* infection (60). To analyze the effect of IL-1R signaling on T cell differentiation during infection, we harvested lungs at serial time points, restimulated the cells with PMA/ionomycin, and stained for intracellular IFNγ, IL-13, and IL-17A as representative cytokines for Th1, Th2, and Th17 polarization states, respectively (Figure 9). The results demonstrated a significantly higher number of CD4^+^ IFNγ^+^ cells in the lungs of WT compared to IL-1RI^−/−^ mice at 7 days postinfection with a trend toward more CD4^+^ IFNγ^+^ cells at days 14 and 21. Compared to the IL-1RI^−/−^ strain, WT mice showed a trend toward more CD4^+^ IL-17A^+^ cells at day 7 with a significant increase of this cell type at days 14 and 21. In contrast, IL-1RI^−/−^ lungs contained a significantly higher percentage of CD4^+^ IL13^+^ cells compared to WT lungs at 14 and 21 days postinfection. In summary, these findings demonstrate that after *C. neoformans* infection, IL-1RI signaling significantly increased Th1 differentiation during the early phase of infection and strongly promoted Th17 differentiation during the late phase of infection.

## Discussion

Induction of IL-1α/β during mouse cryptococcal infection has been reported, but a clear role for IL-1R-dependent signaling in the host immune response has not been demonstrated (27, 32, 34, 35, 37). Here, we provide evidence that IL-1RI deficiency on the BALB/c background has deleterious effects on the outcome of pulmonary *C. neoformans* 52D infection. The most significant findings of this study are as follows: (1) IL-1RI^−/−^ mice cannot clear moderately virulent *C. neoformans* 52D and develop progressive infection of the lungs and brain resulting in death starting at day 40 postinfection; (2) susceptibility of IL-1RI^−/−^ mice is associated with reduced levels of pro-inflammatory, Th1, and Th17 cytokines; (3) IL-1RI signaling in response to *C. neoformans* 52D infection regulates the recruitment of inflammatory DCs to the lung, contributes to recruitment and M1 polarization of macrophages, and promotes Th1/Th17 differentiation of CD4^+^ T cells; and (4) lung neutrophil recruitment associated with IL-1R signaling is dispensable for protection against *C. neoformans* 52D infection. Taken together, these data clearly demonstrate that IL-1R-dependent signaling plays a complex and essential role in the control of progressive *C. neoformans* 52D infection.

Previously, intranasal infection of C57BL/6 and IL-1RI^−/−^ mice with 2 × 10^4^ CFU of the virulent *C. neoformans* H99 strain was shown to cause >90% mortality in both groups (37). In the same report, mice lacking MyD88, an intracellular adaptor for IL-1RI, IL-18R, and several Toll-like receptors, had a trend toward reduced survival but no significant difference in fungal burden compared to WT mice after *C. neoformans* challenge (37). Notably, two earlier studies showed that MyD88^−/−^ mice have a significantly shorter survival time and a higher lung fungal burden compared to WT, TLR2^−/−^, and TLR4^−/−^ mice after *C. neoformans* infection (61, 62). These differences may be attributable, at least in part, to variation in the experimental methods that were used including the dose, route, and strain of *C. neoformans* (60, 63–65). Furthermore, inbred mouse strains also display marked differences in resistance or susceptibility to a standardized cryptococcal infection, highlighting the importance of the host genetic background in disease pathogenesis (66–68). Our data are consistent with other studies showing that BALB/c mice have a naturally resistant phenotype after respiratory infection with the moderately virulent *C. neoformans* 52D strain. Specifically, BALB/c mice progressively clear pulmonary *C. neoformans* 52D infection in association with numerous hallmarks of a protective Th1 response including tight mononuclear cell infiltrates and classically activated macrophages and do not develop central nervous system dissemination (18, 66, 69, 70). Our observation that both IL-1α and IL-1β were induced in the lungs of BALB/c mice after intratracheal infection with *C. neoformans* 52D is also consistent with earlier reports that associated the induction of IL-1β in lung and brain with resistance to cryptococcal infection (35, 71, 72).

Interleukin-1 is a central mediator of inflammation and links innate and adaptive immune response mechanisms (73). Binding of IL-1α or IL-1β to IL-1RI is followed by the recruitment of the IL-1 receptor accessory protein (IL-1RAcP) and activation of signal transduction pathways that induce the expression of IL-1 responsive genes including IL-6, MCP-1, and TNFα (74–77). Induction of pro-inflammatory cytokines followed by generation of a Th1 adaptive immune response is critical for control of cryptococcosis (8, 11, 78). Compared to the BALB/c strain, IL-1RI^−/−^ mice had significantly reduced expression of KC, TNFα, and MCP-1 that was associated with increased lung fungal burden at day 7 after infection. TNFα is one of the main target genes of the IL-1 signaling cascade (76, 77), and both mediators share downstream pathways that induce pro-inflammatory gene expression (79, 80). TNFα signaling in the afferent phase of cryptococcal infection is associated with optimal DC activation and induction of Th1/Th17 polarization and protective immunity (78, 81–84). MCP1/CCR2 signaling is also responsible for the recruitment of inflammatory DCs and macrophages after cryptococcal infection (45, 46, 85). Thus, the reduced expression of pro-inflammatory cytokines and chemokines is one mechanism that could explain the susceptibility of IL-1RI^−/−^ mice to progressive cryptococcosis.

After *C. neoformans* infection, DCs phagocytose and kill cryptococci by oxidative and non-oxidative mechanisms, play an important role in antigen presentation, and drive protective immune responses by secreting cytokines and chemokines (86–89). Compared to other innate cell types, lung DCs express a high level of IL-1RI and signaling *via* this receptor has been shown to promote the maturation and survival of pulmonary DCs and their CCR7-dependent migration to lymph nodes after Influenza A infection (90). At 21 days postinfection with *C. neoformans*, the total number of moDCs in the lung was significantly lower in IL-1RI^−/−^ compared to WT mice, suggesting that recruitment and activation of DCs in the LALNs may be regulated by IL-1R signaling in this model. In addition to DCs, inflammatory macrophages that strongly express microbicidal enzymes such as iNOS play a significant role in fungal clearance (45, 46, 69, 91). After *C. neoformans* 52D infection, we observed that lung macrophages of IL-1R^−/−^ mice had reduced expression of the classical activation markers CD80 and iNOS and increased expression of the alternative activation marker CD206 compared to WT, a pattern that is associated with reduced fungal killing capacity. Our findings are similar to a recent study in BALB/c mice infected with *C. neoformans* 52D that correlated an elevated ratio of Arg1/iNOS expression with an increase in fungal burden and showed a reversal of this ratio during the subsequent period of fungal clearance (48).

In addition to monocyte-derived macrophages and DCs, significantly greater neutrophil recruitment was observed in WT compared to IL-1R^−/−^ lungs. Both IL-1α and IL-1β can promote neutrophil migration (92–95), and diminished neutrophil recruitment to the site of infection due to IL-1R deficiency has been associated with increased susceptibility to several bacterial and fungal infections including *Legionella pneumophila*, Group B *Streptococcus, Citrobacter rodentium*, and *Candida albicans* (55, 96–100). Inbred mouse strains including SJL/J, CBA/J, and BALB/c are naturally resistant to pulmonary cryptococcal infection and exhibit substantial neutrophil recruitment the lungs; however, the importance of these cells in host protection is not clear (35, 67, 68). For example, an early study of BALB/c mice given a single injection of anti-Gr-1 (anti-Ly6C/6G) antibody showed less inflammatory damage and significantly longer survival compared to controls after *C. neoformans* 52D infection (101). A subsequent study of BALB/c mice that had undergone prior immunization with *C. neoformans* strain H99γ showed that neutrophil depletion with a specific anti-Ly6G antibody did not affect pulmonary fungal burden (102). Finally, a recent report showed that profound neutrophilia in type 2-deficient STAT6^−/−^ mice on a C57BL/6 background was associated with immunopathology and exacerbation of cryptococcal disease (103). To specifically analyze the contribution of neutrophils to resistance against *C. neoformans* 52D, we used anti-Ly6G to deplete these cells in WT BALB/c mice throughout the course of infection (104, 105). In the absence of neutrophil recruitment, we observed a significantly lower lung fungal burden at 12 days postinfection compared to controls. This finding suggests that, despite their abundance in the lung, neutrophils may have a detrimental effect on host defense against moderately virulent *C. neoformans* 52D (101). Several mechanisms may explain this observation, including competition for cryptococcal antigen between neutrophils and antigen-presenting cells, neutrophil secretion of the immunosuppressive cytokine TGFβ1, or production of IL-1 receptor antagonist, a molecule that inhibits IL-1R signaling (100, 106–110). Further research is necessary to precisely establish the physiological mechanisms that control neutrophil recruitment during cryptococcal infection and to determine whether these cells make a positive contribution to host resistance.

Along with reduced pro-inflammatory cytokines, IL-1R^−/−^ mice showed diminished levels of lung IFNγ compared to WT mice at the early (day 7) and late (days 14 and 21) phases of infection. Intracellular cytokine staining identified CD4^+^ lymphocytes as the most prominent IFNγ-producing cell type. As very few studies have identified IL-1R expression on Th1 cells (111), induction of IFNγ expression by CD4^+^ T cells appears to be an indirect effect of IL-1RI signaling on DCs and possibly other cell types (90). IFNγ plays a central role in host defense against cryptococci by enhancing the fungal internalization and killing by phagocytes (78, 83). An important role for early IFNγ secretion and the development of a Th1 response against *C. neoformans* 52D infection was previously shown in resistant C.B-17 mice (a BALB/c strain congenic for C57BL/6 immunoglobulin heavy chain gene segment), whereas the absence of this response in the C57BL/6 strain correlated with susceptibility (11).

IL-1 is known to regulate the expression of the transcription factors IRF4 and RORγt, both of which play a major role in the induction of CD4^+^IL-17^+^ (Th17) cells in mice and humans (112–114). IL-1 signaling has been shown to be essential for the development of Th17 immunity to infection with *Coccidioides sp* (115), and mice with deletions of IL-17 or IL-17R are susceptible to candidiasis, pulmonary aspergillosis, and histoplasmosis (55). The role of IL-17 during cryptococcal infection has been analyzed using mice with a C57BL/6 genetic background. In one study, IL-17RA deficiency did not impair pulmonary clearance of *C. neoformans* 52D at 1 or 6 weeks postinfection nor did it alter survival compared to WT mice (116). Another study using IL-17A-deficient mice showed that this cytokine does contribute to fungal clearance from the lung but was not essential for 8-week survival (19). In contrast, administration of IL-23, which is essential for the differentiation of Th17 lymphocytes, led to prolonged survival and reduced fungal burden in C57BL/6 mice (22). A Th17-polarized immune response appears to facilitate the resolution of *C. neoformans* 52D infection through several mechanisms including lung recruitment of activated DCs and inflammatory macrophages, induction of IFNγ-producing CD4^+^ and CD8^+^ T cells, and enhanced fungal containment within macrophages (19–22). Compared to BALB/c, IL-1R^−/−^ mice display several phenotypes that may be attributable to a diminished Th17 response including reduced recruitment of DCs and inflammatory macrophages and increased recruitment of eosinophils and CD4^+^IL-13^+^ cells to the lungs. On the basis of marked difference between WT and IL-1R^−/−^ mice, we speculate that IL-17 plays a non-redundant role in survival after *C. neoformans* 52D infection; however, studies of BALB/c mice that are deficient for IL-17 or IL-17RA would be required to formally test this hypothesis.

In mouse models, IL-1 signaling is protective against infection with a wide spectrum of intracellular pathogens including *Leishmania amazonensis, Mycobacterium avium, Toxoplasma gondii*, and *Listeria monocytogenes* (117–121). IL-1RI-deficient mice are also highly susceptible to pulmonary challenge with *Aspergillus fumigatus*; in this model, IL-1α has been shown to be crucial for optimal leukocyte recruitment and IL-1β has been shown to be essential for optimal activation of macrophage antifungal activity (122). It has been suggested that polymorphisms in the IL-1 gene cluster might be important in susceptibility or resistance to invasive pulmonary aspergillosis in humans (123, 124). Both IL-1α and IL-1β have also been shown to play an important role in disseminated candidiasis (125–128), and IL-1 signaling has shown to contribute to host resistance against pulmonary histoplasmosis and *Coccidioides sp*. infection (115, 129). This study expands the role of IL-1 in host defense by demonstrating that IL-1R^−/−^ mice on the Balb/c background are highly susceptible to progressive *C. neoformans* 52D infection of the lungs and brain. IL-1R deficiency in this model results in impaired Th1/Th17 responses and the development of a Th2-biased adaptive immune response. As IL-1α and IL-1β are equally potent activators of IL-1RI signaling yet have different tissue distribution and activation kinetics, future studies that characterize mice that are deficient in either IL-1α or IL-1β or the study of specific cytokine-deficient animals would provide valuable insights into the specific contributions of each cytokine to the development of protective immunity against *C. neoformans* infection.

## Ethics Statement

This study was carried out in accordance with the recommendations of the Canadian Council on Animal Care guidelines. The protocol was approved by the McGill University Animal Care Committee.

## Author Contributions

MS conceived and performed experiments and wrote/edited the manuscript for important intellectual content. BR and IA performed experiments and edited the manuscript for important intellectual content. DS provided reagents and edited the manuscript for important intellectual content. SQ conceived, designed, and supervised the study and wrote/edited the manuscript for important intellectual content.

## Conflict of Interest Statement

The authors declare that the research was conducted in the absence of any commercial or financial relationships that could be construed as a potential conflict of interest.
